# Understanding the delivery technology used in ADHD stimulant medications can help to individualize treatment

**DOI:** 10.1017/S1092852925000070

**Published:** 2025-02-13

**Authors:** Andrew J. Cutler, Jacob Hanaie

**Affiliations:** 1 Chief Medical Officer, Neuroscience Education Institute, Carlsbad, CA, USA; 2 Department of Psychiatry, SUNY Upstate Medical University, Syracuse, NY, USA; 3 Department of Psychiatry, Kedren Community Mental Health, Los Angeles, CA, USA

**Keywords:** ADHD, delivery technology, individualized treatment, stimulant medications, extended release

## Abstract

Attention-deficit/hyperactivity disorder (ADHD) is a highly heterogeneous disorder for which treatment personalization is essential. Stimulant medications are the first-line option for ADHD management due to their high efficacy. While the choice of stimulant is limited to methylphenidate or amphetamine, there are numerous formulation options each associated with different potential benefits and restrictions. The goal is to deliver a stimulant medication that provides an even, continuous control of symptoms tailored to the patient’s symptoms and lifestyle. This article reviews the technologies used to deliver stimulants and the impact their characteristics have on the pharmacokinetic profiles, dosing regimens, and flexibility of the medication. The aim was to help clinicians in their treatment decision-making process and provide patients with effective and individualized management of their ADHD symptoms.

## Clinical implications

1. A universal approach to treat attention-deficit/hyperactivity disorder (ADHD) is unlikely to be optimal for all patients due to the heterogeneity in etiology, neurobiology, symptoms, impairment, comorbidities, and disease course.
2. There are a limited number of therapeutic categories of medication to treat this highly variable condition; and the technology used to deliver stimulants, the first-line medical management option, becomes more relevant as clinicians look to personalize treatment.
3. The most desired features of stimulant medication are a quick onset of efficacy, a long and consistent duration, minimal interactions with concomitant medications or food, reduced abusability, and a smooth pharmacokinetic curve without a sharp peak or an abrupt offset.
4. ADHD delivery technologies impact the pharmacokinetic profile by altering the timing of drug release and the plasma concentrations achieved over the course of the day, which influence the clinical onset and duration as well as tolerability of the medication.
5. This review discusses the benefits and restrictions of the various stimulant delivery technologies to help guide healthcare providers in their ADHD treatment choices.

## Introduction

Attention-deficit/hyperactivity disorder (ADHD) is a neurodevelopmental disorder characterized by inattention, hyperactivity, and impulsivity.^1^ Since ADHD is heterogeneous in etiology, neurobiology, symptoms, impairment, comorbidities, and disease course,^2^ a universal approach to treat ADHD is unlikely to be optimal for all patients. Considerations that impact treatment decisions include the patient’s response to current or previous medication, the onset and duration of coverage for which symptom relief is required, concomitant medications and their effects on the patient and/or other medications, present comorbidities, patient preference, and the likelihood of adherence to different formulations.^3^^,^^4^ With the substantial neurobiological and clinical variability associated with ADHD and the limited number of therapeutic categories of medication available for treatment, the technology involved in the delivery of these medications becomes more relevant for their effective utilization.

This article focuses on the delivery technologies typically used in stimulant medications for ADHD, as stimulants are the first-line treatment option in ADHD management due to their high efficacy.^2^ Amphetamine (AMP) and methylphenidate (MPH) are the two types of stimulants approved in the United States (US) to treat ADHD. Both are short-acting, and different formulations contain one of the isomers or a racemic mixture.^5^ As ADHD symptoms and impairments often persist all day long beyond work or school hours, there has been an evolution in extended-release (ER) technologies to provide all-day coverage, and several formulations of long-acting stimulants have received approval in recent years. It is important to understand the characteristics of the technologies used, their clinical relevance, and the resulting impact on treatment outcomes. This article presents the technologies and formulations that were developed in response to the evolving needs and understanding of the ADHD disease state, highlighting the advantages and limitations of these solutions.

New formulations possess unique advantages to accomplish the common goals of a stimulant—to provide a quick onset of efficacy, a long and consistent duration, minimal interactions with medications or food, reduced abusability, and a smooth pharmacokinetic (PK) curve, avoiding a sharp peak or an abrupt offset. In 2019, the US Food and Drug Administration (FDA) issued a Guidance for Industry on developing stimulants for ADHD, which stated that stimulants have a strong concentration–response relationship for efficacy and safety.^6^ This refers to the relationship between the PK (plasma level, or the movement of drugs in the body) and pharmacodynamics (PD; clinical effect) of the drug. ADHD delivery technologies impact the PK profile by influencing the timing of drug release and the plasma concentrations achieved over the course of the day, which in turn translate into the clinical onset and duration as well as tolerability of the medication.^7^ Additionally, there are PK characteristics that should be considered when translation to efficacy is the goal,^6^ for example, one should not just consider threshold levels but ascension to the threshold as an important part of the efficacy story.^6^ Due to different properties, some formulations have demonstrated undesirable inter- and intraindividual variability in clinical effects,^8^ and therefore, stimulants have to be individualized for optimal efficacy and tolerability. Notably, the metabolism of both methylphenidate and amphetamine may differ between individuals due to genetic variability in the enzymes that metabolize them^8^ and/or dopaminergic tone or response.^9^

An increased understanding of the technologies used in stimulant medications can assist clinicians in tailoring ADHD treatment to patients’ needs. The following review covers the evolution of long-acting stimulant formulations and the various technologies involved, with a discussion based on the authors’ clinical experience in using the different technologies to guide treatment individualization and optimization for their patients.

## Development of delivery technologies

The first stimulant medications to treat ADHD were immediate-release (IR) formulations of amphetamine or methylphenidate. However, due to the short duration of action, patients required multiple doses each day. This has led to poor adherence^10^^,^^11^ and significant fluctuations in medication levels in the blood over the course of the day, which can manifest clinically as side effects at the peak plasma level, a rapid decrease in efficacy, or a rebound or “crash” syndrome (cognitive impairment, irritability, emotional lability, etc.) due to sharp decline in plasma level.^7^ Early technologies were developed to mimic twice-a-day (BID) dosing, which was the standard at the time. It was subsequently realized that a smoother PK curve with a broader peak and a gradual descent in plasma levels was preferable.

### Osmotic release

One of the first technologies developed to extend therapeutic duration was osmotic release, including OROS® (osmotic-controlled release oral system) used in Concerta® and Osmodex® used in Relexxii®. Osmotic delivery systems utilize both an overcoat of IR methylphenidate that is absorbed immediately to provide an early onset of efficacy and an ER component dependent on osmotic pressure. Tablets have multiple chambers that either contain medication or serve as an inert “push” compartment. As medication travels through the gastrointestinal (GI) tract, water is absorbed by an osmotic membrane in the “push” chamber, causing it to swell and push the medication out of the tablet through a small laser-drilled hole.^12^ This technology successfully extended the duration of efficacy, allowing for once-daily dosing. However, at the time of development, a tachyphylaxis phenomenon was noted during the day, which may or may not have been clinically relevant, resulting in an attempt to provide ascending plasma levels from the first to the second peak.^13^ Utilizing two asymmetric release points is one of the several limitations that persist with osmotic delivery,^12^ which could result in undesirable variability in plasma medication levels throughout the day. In fact, the OROS® technology was designed to deliver 22% of the total methylphenidate via the IR coating and 78% via osmotic delivery.^12^ As there is a close relationship between the PKs of a stimulant and its clinical effect,^6^ this could result in delay and variability in efficacy throughout the day.

Additionally, the controlled-release tablet does not deform—it must be swallowed whole and cannot be given to patients with severe GI narrowing.^12^

### Multiple beads

**Figure 1.**
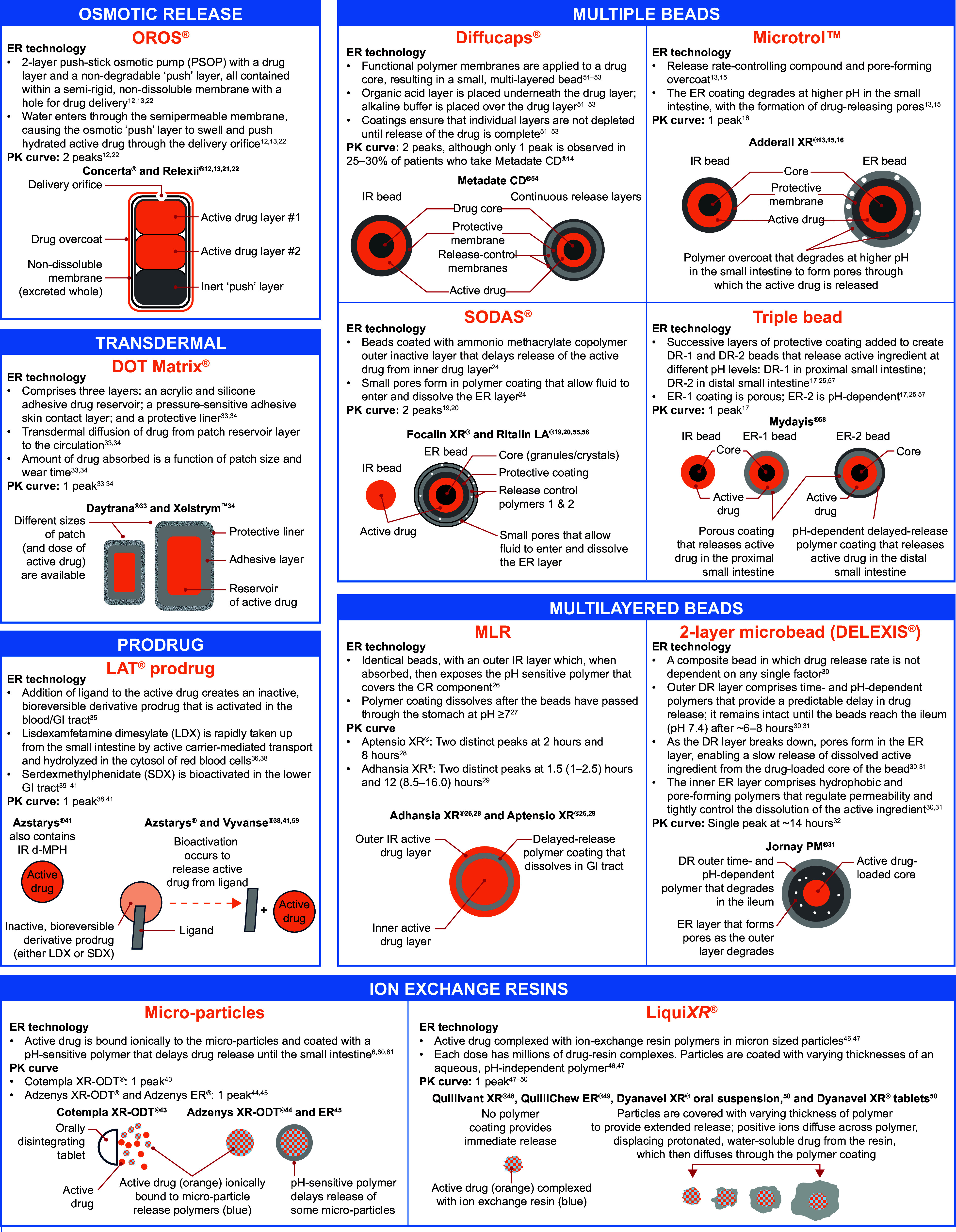
A summary of the ER/DR technologies available for stimulant medications in ADHD. ADHD, attention-deficit/hyperactivity disorder; CR, controlled release; d-MPH, dexmethylphenidate; DR, delayed release; ER, extended release; GI, gastrointestinal; IR, immediate release; LDX, lisdexamfetamine; MLR, multilayer release; OROS, osmotic-controlled release oral system; PK, pharmacokinetic; SDX, serdexmethylphenidate; SODAS, spheroidal oral drug absorption system.

Beaded technologies emerged as an alternative ER delivery system. Beaded systems using multiple different beads in one capsule often leverage the changing pH throughout the GI system to direct the release of medication at distinct locations to give IR and ER. The first iteration of ER medication utilizing this technology, Diffucaps® used in Metadate CD® (currently only available as a generic, methylphenidate HCl ER tablet), allowed for once-daily dosing.^14^ In this medication, 30% of the methylphenidate is delivered with the IR bead, and 70% is delivered by the ER bead.^14^ However, like the PK curve seen with BID IR dosing and osmotic release, this long-acting formulation continued to show peaks and troughs in blood levels across the day.^14^ The Microtrol™ technology^15^ used in Adderall XR®^16^ similarly delivers mixed amphetamine salts using two populations of beads.^7^ The first is an IR bead designed to release medication in the stomach (pH 1.5–3.5), and the second is an ER bead that releases in the small intestine (at pH 5.5).^7^^,^^15^ Unlike Diffucaps®, the Microtrol™ technology delivers mixed amphetamine salts in an equal 50:50 split of IR and ER between the two beads, which provides a smoother PK curve. However, clinicians found the duration of efficacy to be too short for some patients, particularly adults, leading to the development of another formulation used in Mydayis®. This medication adds a third bead that releases medication even more distally in the intestine (at pH 7), thus potentially extending the efficacy to up to 16 hours.^17^ Amphetamine is delivered in an equal percentage of ⅓:⅓:⅓ between the three beads.^18^ Another technology, the spheroidal oral drug absorption system (SODAS®), is used in Focalin XR® and Ritalin LA®. SODAS® also uses two populations of IR and ER beads with an equal 50:50 split in methylphenidate delivery.^19^^,^^20^ Please refer to Table 1 and Figure 1 for specific compositions of the technologies mentioned.

**Table 1.** Overview of drug delivery technologies and the brands that use those technologies to provide extended-release formulations of stimulant medications for the treatment of ADHD. The PK parameters are those that are easily available from the prescribing information (PI) of each product

Delivery system technology	Technology components	Mechanism of release	Example product(s)	PK features (healthy adults)			Specific dosing instructions	Clinical considerations
				Release profile	Tmax (hours)	T½ (hours)		
Osmotic release								
OROS®	2-layer push-stick osmotic pump (PSOP), comprising two drug reservoirs and an IR outer layer “Push” layer comprises osmopolymers to control the rate of active drug release The layers are surrounded by a semirigid, nondissoluble membrane with a precision laser-drilled hole for drug delivery ^13^^,^^21^	A controlled rate of water entry through a non-degradable, semipermeable membrane; causes swelling of the osmotic layer and hydrates active drug The swollen push layer controls the rate of hydrated active drug release through the delivery hole at the same rate as water entry ^10^^,^^21^	Concerta® MPH tablets ^12^	Two peaks	18 mg dose 6.8 ± 1.8 (A)	3.5 ± 0.4 (A)	Must be swallowed whole with liquid and not chewed, divided, or crushed	Should not ordinarily be used in patients with preexisting severe pathologic or iatrogenic gastrointestinal narrowing The tablet shell and insoluble core components are eliminated whole in the stool
			Relexxii® MPH tablets ^22^	Two peaks	18 mg dose 6.8 ± 1.8 (A)	3.5 ± 0.4 (A)	Must be swallowed whole with liquid and not chewed, divided, or crushed	Should not ordinarily be used in patients with preexisting severe pathologic or iatrogenic gastrointestinal narrowing The tablet shell and insoluble core components are eliminated whole in the stool
Multiple beads								
Diffucaps®	Release-controlling polymer membranes are applied to a neutral core in multilayered beads of <1.5 mm diameter Each dose may include a mixture of beads with different levels of coating or uniform multilayered beads to achieve the desired IR, ER, or DR dissolution ^23^	Beads comprise active drug, excipients, and release-controlling polymers pH-dependent layers control the bead microenvironment to ensure drug solubility and dissolution for the desired PK profile ^23^	Metadate CD® MPH capsules for pediatric patients only ^14^	Two peaks	20 mg dose Fa: Tmax1: 1.5 Fa: Tmax2: 4.5	6.8	Take whole or on applesauce*	25–30% of children had only one MPH peak A high-fat meal increases Cmax by ~30% and Tmax1 by ~1 hour Take before breakfast with liquid
Microtrol™ (2 bead)	IR and ER beads IR beads comprise an inert core + active drug, with/without excipient ER beads comprise IR beads plus release rate-controlling compound and pore-forming overcoat ^13^^,^^15^	IR beads begin to release drugs immediately after ingestion The coating of the ER beads gradually degrades (forming drug-releasing pores) in the higher pH (~5.5) of the small intestine ^13^^,^^15^	Adderall XR® MAS 30 mg capsules ^16^	Single peak	30 mg dose Fe *d*-AMP: 7.7 Fe *l*-AMP: 8.3 Fa *d*-AMP: 5.2 Fa *l*-AMP: 5.6	*d*-AMP: 10 (A); 11 (AD); 9 (C) *l*-AMP: 13 (A); 13–14 (AD); 11 (C)	Take whole or on applesauce*	Food does not affect the absorption of *d*- and *l*-AMP but prolongs Tmax by 2.5 hours Tmax decreased when given with a PPI Coadministration with GI or urinary alkalinizing agents should be avoided
SODAS® (Spheroidal Oral Drug Absorption System)	Uniform spherical nonpareil beads of 1–2 mm in diameter with ammonio methacrylate copolymer outer sealant to delay release of active drug ^24^	ER beads coated with an inactive, pore-forming layer to delay release of active drug from inner drug layer ^24^	Focalin XR® d-MPH capsules ^19^	Two peaks	20 mg dose (A) Tmax1: 1.5 (1–4) Tmax2: 6.5 (4.5–7)	~3 (A) 2–3 (C)	Take whole or on applesauce*	
			Ritalin LA® MPH capsules ^20^	Two peaks	20 mg dose Tmax1: 2.0 ± 0.9 (1.3–4.0) (A); 2.0 ± 0.8 (1–3) (C) Tmax2: 5.5 ± 0.8 (4.3–6.5) (A); 6.6 ± 1.5 (5–11) (C)	3.3 ± 0.4 (3.0–4.2) (A) 2.4 ± 0.7 (1.5–4.0) (C)	Take whole or on (not warm) applesauce*	A high-fat meal decreases Cmax2 by 25%, and there are variable delays in Tmax1 and Tmax2 Administration times relative to meals and meal composition may need to be individually titrated
Triple bead	IR beads comprise 0.5–0.6 mm inert sugar spheres coated with active drug Successive layers of protective coating are added to create two delayed-release layers ^17^^,^^25^	The three bead types release active drugs at different pH levels, enabling prolonged delivery over time as the pH in the GI tract changes: IR in the stomach; DR–1 at pH 5.5 (proximal small intestine); DR–2 at pH 7.0 (distal small intestine) ^17^^,^^25^	Mydayis® MAS capsules ^17^	Single peak	50 mg dose Fe *d*-AMP: 12 Fe *l*-AMP: 12 Fa *d*-AMP: 7 Fa *l-*AMP: 7.5	*d*-AMP: 10–11 (A, C) *l*-AMP: 10–13 (A, C)	Take whole or on applesauce* Administer only upon awakening due to the potential for causing insomnia	Patients ≤12 years show higher plasma exposure and rates of AEs, at the same dose, as those ≥13 years Advise patients to take consistently either with or without food A high-fat meal increases Tmax by 4.5–5 hours Monitor for changes in clinical effect with concomitant gastric pH modulator and use alternative therapy based on clinical response
Multilayered beads								
MLR	Nonpareil ~1 mm spheres with an IR layer and a polymer-coated CR layer ^26^	Active drug is released from the CR layer as the polymer coating dissolves in the GI tract ^27^	Aptensio XR™ MPH capsules ^28^	Two peaks	80 mg dose Fa Tmax1: 2 (capsule/sprinkled) Fa Tmax2: ~8 (capsule/sprinkled)	5.09 (capsule); 5.43 (sprinkled)	Take whole or on applesauce*	Establish a routine for administration relative to meals A high-fat meal increases Cmax by ~28% and decreases Tmax2
			Adhansia XR® MPH capsules ^29^	Two peaks	100 mg dose Fa: Tmax1: 1.5 (1–2.5) Fa: Tmax2: 12 (8.5–16)	7	Take whole or on applesauce*	A high-fat meal increases Tmax (for both Cmax values) by ~1 hour Monitor for changes in clinical effects with concomitant gastric pH modulator and use alternative therapy based on clinical response
2-layer microbead (DELEXIS®)	Outer DR layer comprises time- and pH-dependent polymers to provide a predictable delay in drug release, controlled by ratio of components that affect wettability and rate of erosion Inner ER layer comprises hydrophobic and pore-forming polymers to regulate permeability and control the dissolution of active drug ^30^^,^^31^	DR layer remains intact until the beads reach the ileum (pH 7.4) ~6–8 hours after ingestion As the DR layer breaks down, pores form in the ER layer, enabling a slow release of dissolved active drug from the central core ^30^^,^^31^	Jornay PM® MPH capsules ^32^	Single peak	100 mg dose Fa: 14 (A)	Fa: 5.9 (A)	Requires a routine administration time, consistently with/without food, whole or on applesauce* Must be taken 10 hours before required effect; cannot be taken in the morning if forgotten in the previous evening	A high-fat meal at night decreases Cmax by 14% and extends Tmax by 2.5 hours
Transdermal patch								
DOT Matrix® Transdermal: skin patch	Comprises three layers: acrylic and silicone adhesive drug reservoir, pressure sensitive adhesive skin contact layer, and protective liner ^33^^,^^34^	Active drug diffuses transdermally from the reservoir layer of the patch to the circulation The amount of drug absorbed is a function of patch size and wear time ^33^^,^^34^	Daytrana® MPH transdermal patch ^33^	Single peak	12.5–37.5 cm^2^ patch Single use: ~10 Repeat exposure: 8	4–5 hrs after patch removal (for patients aged 6–17 years)	Apply ~2 hrs before effect needed Remove at <9 hours to reduce the duration of effect or in case of side effects Absorption of *d*-MPH may continue for several hours after patch removal	Use alternating hip sites that are not oily, damaged, or irritated Direct external heat can increase Tmax by 0.5 hours and increase Cmax by 2-fold When applied to inflamed skin, lag time is ≥1 hour, Tmax is 4 hours, and both Cmax and AUC are ~3-fold higher May cause skin reactions; monitor for signs of skin pigmentation
			Xelstrym™ *d*-AMP transdermal patch ^34^	Single peak	18 mg/9 hours dose Single application: 6–9 Repeat applications: 6	Patch removal after 9 hrs wear time: 11.5 (A); 6.4 (C)	Apply ~2 hrs before effect is needed Avoid direct external heat sources as they can increase the rate and extent of absorption of AMP by x1.5	Application of a heating pad for 6 consecutive hours increases Tmax to ~6.5 hours Select a different application site (clean, dry, intact skin) each day to limit the occurrence of skin reactions
Prodrug								
LAT® prodrug	Ligand is added to the active drug molecule to create an inactive, bioreversible derivative prodrug that is activated after oral administration of the tablet ^35^ Prodrug has no or limited pharmacological activity ^35^	LDX is rapidly taken up from the small intestine by active carrier-mediated transport ^36^ Hydrolysis of LDX to release d-AMP occurs in the cytosol of red blood cells, which have a high capacity for the metabolism of LDX ^37^	Vyvanse® LDX capsules (pediatric) ^38^	Single peak	30, 50, or 70 mg dose Fa LDX: ~1 (6–12 years) Fa: *d*-AMP: ~3.5 (6–12 years)	LDX: <1 *d*-AMP: 12	Avoid afternoon doses due to the potential for insomnia Take whole or mix the contents with yogurt, water, or orange juice; consume immediately	Food prolongs Tmax by ~1 hour
			Vyvanse® LDX chewable tablets ^38^	Single peak	60 mg dose Fa LDX: ~1 (A) Fa *d*-AMP: 4.4 (A)	LDX: <1 *d*-AMP: ~12	Avoid afternoon doses due to the potential for insomnia Chew thoroughly before swallowing	A high-fat meal decreases Cmax by 5–7% and prolongs Tmax by ~1 hour
		In addition to an IR *d*-MPH component, SDX is bioactivated by serine cleavage in the lower GI tract to release *d*-MPH ^39^^-^^41^	Azstarys® SDX (70%), IR *d*-MPH (30%) capsules ^41^	Single peak	52.3 mg/10.4 mg dose Fa: ~2 hrs (SDX and *d*-MPH)	SDX: 5.7 *d*-MPH: 11.7	Take whole or on applesauce* or water	Presence of food extends Tmax by 2–4.5 hrs
Ion-exchange resins								
Microparticles: cation exchange resin	Each ODT has drug ionically bound to 100 000–200 000 polystyrene sulfonate microparticles The positively charged drug is dissolved in the presence of an exchange resin and replaces the Na+ ion of the resin to form stable drug containing microparticles ^42^	IR portion releases the drug immediately in the gastric lumen Release is delayed until the small intestine for microparticles coated with a pH-sensitive polymer ^42^	Cotempla XR-ODT® MPH orally disintegrating tablets ^43^	Single peak	51.8 mg dose Fa: 5	~4	Take consistently with/without food; allow tablets to disintegrate on the tongue	High-fat meal decreases Cmax by ~24% and Tmax by 0.5 hours Not recommended for concomitant use with a gastric pH modulator
			Adzenys XR-ODT® AMP orally disintegrating tablets ^44^	Single peak	18.8 mg dose Fe: 7 (3–16) (A) Fa: 5 (3–12) (A)	Fe: 11.33 ± 2.0 (A) Fa: 11.25 ± 2.0 (A)	Allow tablet to disintegrate in saliva and then swallow	Food reduces Cmax *d*-AMP by 19% and increases Tmax by 2 hours (*d*-AMP) and 2.5 hours (*l*-AMP)
			Adzenys ER™ AMP oral suspension ^45^	Single peak	18.8 mg dose Fa: *d*-AMP: 5 (3–7.5) (A) Fa*: l*-AMP: 5 (3–8) (A)	*d*-AMP: 11.4 ± 2.3 (A); 12.7 ± 6.4 (C) *l*-AMP: 14.1 ± 3.5 (A); 15.3 ± 14.4 (C)	Shake the bottle before administering	Unaffected by food Not recommended for concomitant use with a gastric pH modulator
LiquiXR®: coated resin complex	Active drug complexed with ion-exchange resin polymers in micron-sized particles Varying thicknesses of polymer coating applied to resin particles Millions of drug-resin complexes in each dose ^46^^,^^47^	IR uncoated particles ER particles coated with varying thicknesses of an aqueous, pH-independent non-dissolving polymer Positively charged ions diffuse across the particle coating to displace protonated, water-soluble drug from the resin ^46^^,^^47^	Quillivant XR® MPH extended-release oral suspension ^48^	Single peak	60 mg dose Fe: 4.04 (1.3–7.3 (A); 2.0 (1.98–4.0) (AD); 4.05 (3.98–6.0) (C)	Fe: 5.2 ± 1.0 (A); 5.0 ± 0.2 (AD); 5.2 ± 0.1 (C)	Reconstitute and vigorously shake for >10 seconds	High-fat meals increase Cmax by 28% and Tmax by ~1 hour
			QuilliChew ER® MPH chewable tablets ^49^	Single peak	40 mg dose Fa 5	Fe: 5.2	20 mg and 30 mg tablets are scored for division into equal halves	High-fat meals have no effect on Tmax but increase Cmax by ~20%
			Dyanavel XR® AMP MPH extended release oral suspension ^50^	Single peak	18.8 mg dose Fa *d*-AMP: 4 (2–7) (A) Fa *l*-AMP: 5 (3–7)	*d*-AMP: 12.4 *l*-AMP: 15.1	Shake well before every dose; use oral dispenser provided	High-fat meals increase Cmax by ~2% and increase Tmax by ~1 hour
			Dyanavel XR® AMP chewable tablets ^50^	Single peak	20 mg dose Fa *d*- & *l*-AMP: 5 (2–9))	*d*-AMP: 13.5 *l*-AMP: 17.3	5 mg tablets are scored for division into equal halves	High-fat meals increase Cmax by ~3% but do not delay median Tmax

*Note:* All products can be taken with or without food unless as described in clinical considerations column; the impact of high-fat meals on Cmax and/or Tmax for methylphenidates is due to variability associated with the active drug combined with the technology.

Abbreviations: A, adults; AD, adolescents; ADHD, attention-deficit/hyperactivity disorder; AE, adverse event; AMP, amphetamine; C, children; Cmax, peak plasma concentration; CR, controlled release; DR, delayed release; d-MPH, dexmethylphenidate; ER, extended release; Fe, fed; Fa, fasted; GI, gastrointestinal; IR, immediate release; LDX, lisdexamfetamine; MAS, mixed amphetamine salts; MLR, multilayer release; MPH, methylphenidate; ODT, orally disintegrating tablet; OROS, osmotic-controlled release oral system; PK, pharmacokinetic; PPI, proton pump inhibitor; SDX, serdexmethylphenidate; SODAS, spheroidal oral drug absorption system; T½, terminal half-life; Tmax, time to peak plasma concentration.

*Take whole or sprinkled on applesauce without chewing.

### Multilayer beads

Other beaded systems use one population of beads with multiple functional layers. Examples of this multilayer-release (MLR) bead technology include Aptensio XR™ and Adhansia XR®. Duration of clinical efficacy was improved between the development of Aptensio XR™ and Adhansia XR®. In Adhansia XR®, the technology of the second release layer was changed to release medication even more gradually to extend the duration of efficacy for adolescents and adults.

Another multilayered technology is DELEXIS®, used in Jornay PM®. DELEXIS® consists of a single population of two-layer microbeads with delayed-release (DR) and ER layers around a drug-loaded core, allowing for evening dosing with onset of efficacy the following morning. In healthy adults, a single 100 mg oral dose given in the evening showed an initial time lag of 10 hours.^32^ As the bead traverses the GI tract, increasing wettability leads to the breakdown of the hard outer DR layer overnight, forming pores that expose the underlying ER layer to GI fluids. The increased wetting of this soluble ER layer causes it to slowly dissolve, allowing for gradual diffusion of the medication from the core into the intestinal lumen, where it is absorbed.^30^^,^^31^ Since colonic absorption is less efficient than that in other regions of the GI tract, it contributes to a smooth rise in plasma medication levels during release, as well as a broad peak concentration and a subsequent gradual descent in plasma levels.^57^^,^^60^ This technology is associated with considerable intraindividual consistency in the time from ingestion to clinical effect.

While the evolution of beaded technologies has allowed for smoother PK curves, extended clinical duration, and flexibility around the time of dosing, the biggest limitation for most of them lies in the potential for factors that influence pH (diet, medications) and GI transit time, thereby altering the release profile.

### Transdermal delivery

A desire for flexible daily dosing and an alternative to oral formulations led to the development of transdermal delivery systems. The DOT Matrix® used in Daytrana® (methylphenidate) and Xelstrym™ (amphetamine) allows patients to adjust medication duration by simply changing the wear time, providing a potentially shortened duration when the patch is peeled off early (although MPH concentrations are reported to decline in a biexponential manner, likely due to continued distribution of MPH from the skin after patch removal). It also provides visual verification of adherence.^34^ This technology offers an option for patients who cannot or prefer not to swallow pills, commonly seen in children or patients with co-occurring autism spectrum disorder.^61^ While transdermal delivery addresses several patient needs, it is limited by a delayed onset of efficacy, dermal side effects such as skin irritation, and increased rate and extent of absorption when applied to inflamed skin.^33^^,^^34^

### Prodrug

Prodrug technology represented a significant advancement in stimulant formulations. A prodrug is an active medication covalently bound to an inactive moiety, which renders the prodrug inactive until it is cleaved by an endogenous enzyme to release the active medication. This gradual process results in a slow release of the medication.^38^ The LAT® prodrug technology first used in Vyvanse®, or lisdexamfetamine (LDX, a molecule of lysine attached to dextroamphetamine), requires absorption in the large intestine and subsequent cleavage of the lysine group in red blood cells to release the medication. Of note, its package insert reports inter-subject variability of 25% or less,^38^ and there could be variation in clinical effect based upon variations of absorption and conversion of LDX.^6^ To overcome this delay in clinical onset of effect,^62^ Azstarys® also has an IR component (dexmethylphenidate) to provide an early onset of efficacy while preserving a smooth extended duration of release.^39^ The prodrug delivery system results in a gradual increase in plasma amphetamine levels, a characteristic believed to confer properties of reduced abuse potential.^9^ Two studies have demonstrated that Vyvanse®, at FDA-approved doses, does not elicit high “likeability” scores from patients with a history of stimulant abuse compared to responses to IR d-AMP.^63^^,^^64^ While the serdexmethylphenidate prodrug component of Azstarys® showed decreased potential for abuse and by itself is a C-IV drug,^65^ the combination with IR dexmethylphenidate renders Azstarys® a C-II drug, like other stimulants.

### Ion-exchange resins

Individual differences in metabolism of amphetamine and methylphenidate^66^^–^^68^ can mediate the duration of clinical efficacy when the medication is released as a bolus, characteristic of beaded and osmotic pump technologies. Ion-exchange resins are formed by dissolving methylphenidate or amphetamine salt. The base drug (a positively charged cation) is separated from the negatively charged anion salt molecule and bound to a negatively charged resin particle. Ion-exchange technology allowed for the development of ER formulations such as liquids, oral dissolving tablets (ODTs), and chewables for patients who cannot or prefer not to swallow pills. While providing a smooth PK profile, fast onset (via IR components), and extended duration (via ER components), microparticle technologies used in Adzenys XR-ODT® and Cotempla XR-ODT® rely on the changing pH throughout the GI tract to mediate medication release.^69^ Therefore, similar to beaded technologies, drug release can be altered by diet- and medication-induced changes to gut pH.

Liqui*XR*® is an advanced ion-exchange and diffusion technology used in Dyanavel XR®, Quillivant XR®, and QuilliChew ER® that addresses this shortcoming by utilizing pH-independent processes. Each dose contains a mixture of ER and IR particles. ER particles are coated with a protective layer that does not dissolve as the medication moves throughout the GI tract. Instead, positively charged ions endogenous to the gut diffuse through the coating and dislodge medication molecules, which then diffuse through the coating into the GI cavity where the medication is absorbed. The coating thickness around particles varies, resulting in numerous release points rather than a bolus-style release; this is reflected in a smooth rise in plasma medication levels and a gradual decline at the end of the day.^47^^–^^50^ Thinner coatings provide a shorter diffusion distance and release molecules of medication sooner in the day, whereas thicker coatings require a longer diffusion path and therefore release the medication later. This unique combination of diffusion coating thicknesses across ER particles achieves a smooth, continuous extended release of the medication, while uncoated particles provide immediate medication release for a fast clinical onset.^70^^,^^71^ The drugs that use Liqui*XR*® technology are among the few branded products still on the market, so access may be limited by their potential expense.

The variety of delivery technologies and progressive sophistication within each class of delivery systems have improved many aspects of the clinical profile of stimulants. Cumulatively, delivery system advancements have provided medication options with early onset and extended duration of efficacy, improved tolerability via decreased fluctuations in plasma levels, allowed flexibility for dose adjustments, provided non-oral formulation options, reduced abuse potential, and limited variability of therapeutic response from individual differences in pH or metabolism. Each delivery system addresses a unique set of challenges faced by patients while carrying its own limitations. It is, therefore, important for clinicians to consider these factors to optimize patient outcomes.

## Discussion

**Figure 2.**
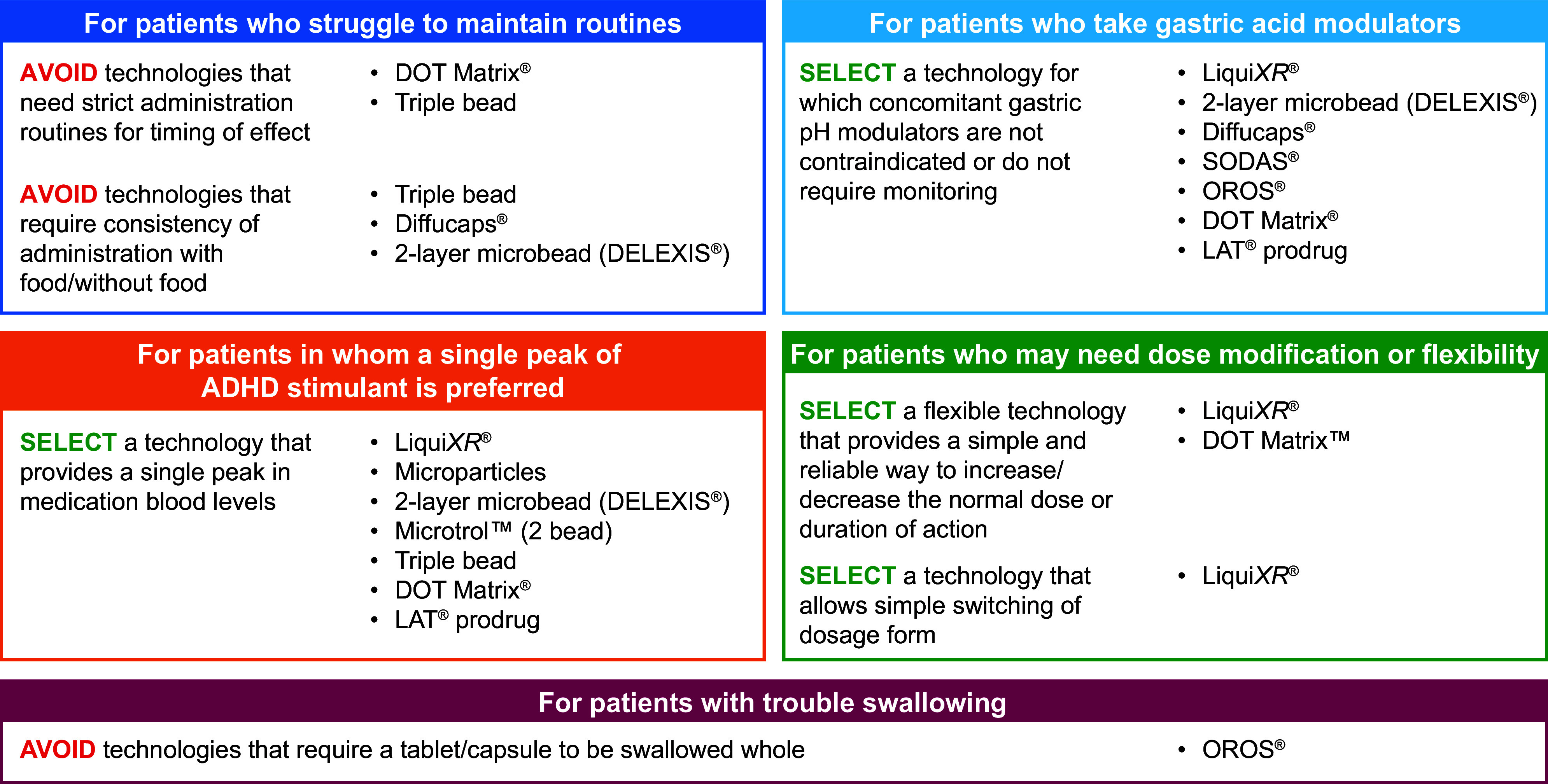
A summary of clinical considerations for ADHD stimulant treatment, based on the characteristics of the different technologies. ADHD, attention-deficit/hyperactivity disorder; OROS, osmotic-controlled release oral system; SODAS, spheroidal oral drug absorption system.

Understanding the technologies used in different ADHD stimulants will better equip clinicians to customize ADHD treatment according to patient needs and preferences (Figure 2). Provided are some examples that demonstrate how various technologies meet patient need.

Some patients may prefer flexibility in dose adjustments or administration. Flexible dosing may be appreciated during initial titration in patients who require small adjustments due to sensitive tolerability or for those anticipating different duration needs. Formulations with ease of adjustment include liquid formulations that allow small dose adjustments, scored tablets which can be halved, and transdermal patches which can be removed early to shorten the duration of exposure. For flexibility of administration, patients who cannot or prefer not to swallow pills can take formulations that are liquid, chewable, sprinkled, orally disintegrated, or transdermal through a patch.

Certain technologies used in ADHD medications require consistency in administration and might not be suitable for all patients. For example, medications that have a delayed onset—including the osmotic delivery system, transdermal patch, and LDX prodrug—may require advanced planning. In the case of the DELEXIS® multilayer bead, if the patient wakes in the morning and realizes that they have missed the dose the night before, they cannot take it until the next scheduled time. Furthermore, patients may need to time their meals for certain medications as food (based on fat content or acidity) can alter drug release in some formulations. Other concerns include the need to avoid coadministration of certain medications or agents that would alter gut pH; for the latter, patients may benefit from medications that release independently of pH.

The timing of medication release differs with the ADHD delivery technologies. Patients may have individual needs for therapeutic onset based on their lifestyle or preferences. For example, the LDX prodrug,^38^ osmotic release,^12^^,^^22^ and transdermal patch^33^^,^^34^ technologies have a delayed drug release and subsequent delay in providing efficacy.^6^ If a patient commutes to school or works shortly after waking and taking medication, they may prefer an alternative formulation with a more rapid effect. Moreover, patients may have different needs when it comes to the duration of effect, which can also be matched to appropriate formulations and delivery technologies, for example, adjusting the timing of effect to reduce appetite suppression around mealtimes or choosing ER formulations with a shorter duration of efficacy might suit younger patients or those with shorter school or work days. The proportion of IR and ER components used in different technologies is an important consideration when selecting medication for a desired onset and duration. For example, osmotic release has a higher proportion of ER, whereas Microtrol™ beaded technology uses a 50/50 split of IR and ER components.

Some patients report that they like feeling their medication start working, while many prefer a smooth on and off. Technologies to smooth the PK peaks include the 2-layer microbead (DELEXIS®), in which the delayed absorption in the colon translates into less pronounced peaks than those relying on absorption earlier along the GI tract, and Liqui*XR*®, which contains numerous particles with variable coating thicknesses to consistently deliver the active drug. Other patients may prefer the release of medications in boluses, perhaps to regain appetite during parts of the day when plasma levels dip. Formulations with a smoother PK curve, and especially with a slower decline in plasma levels, may contribute to a more gradual offset of efficacy and lower risk of rebound and crash. Furthermore, immediate-release formulations can be used as needed or as supplementation to an ER or transdermal formulation to extend the duration of action.

## Conclusion

Medications, particularly stimulants due to their significant efficacy, are an important component in the management of ADHD. Numerous variables need to be considered when evaluating the optimal choice to treat ADHD patients. The technology of stimulant formulations has evolved over time, as has our understanding on how ADHD stimulants should be administered—from early formulations which mimicked multiple IR dosing daily, with or without an ascending peak, to the current goal of providing a smooth, consistent level of stimulant release for the duration of the dose, with rapid onset and gradual offset of action.

The key outcome in the management of ADHD is the level of symptom control during a dosing period. Since plasma levels of ADHD stimulants translate into clinical effect, different drug delivery technologies can affect the concentration of stimulant in the blood and impact how well the medication controls symptoms. The timing of medication release by delivery technologies as well as the proportion of IR and ER components determine the resulting onset and duration of efficacy. Furthermore, the nature of drug release, as distinct boluses or a continuous pattern, results in distinct patterns of plasma levels and thus symptom control over the day. Technologies that rely on certain factors for drug release, such as pH, can be affected by external factors including medications or diet, and these interactions can have clinical consequences.

The management of ADHD requires numerous considerations to match the clinical needs, lifestyle, and social circumstances of each patient.^4^ The different formulation technologies of stimulants present clinicians with a wide range of options from which the most appropriate treatment—based on symptom presentation, dosing interval, route of administration, concomitant medications, patient preferences, and lifestyle—can be selected. Ultimately, cost and access dictate many of our treatment choices, but for most patients, the newer formulations with more desirable PK profiles that provide a faster onset, smoother PK curve, longer duration, and more gradual decrease at the end of the day (less crash/rebound) are desirable.
